# 918 Utilizing Advanced Practice Providers in a Non-Academic Burn Center Increases Productivity and Accommodates Growth

**DOI:** 10.1093/jbcr/iraf019.449

**Published:** 2025-04-01

**Authors:** Brett Bird, Alison Spatz, Lindsay Desantis, Laurin Proctor, Samantha Allbritton, Lily Daniali, Ryan Endress, Wojciech Przylecki, Benson Pulikkottil

**Affiliations:** HCA HealthONE Swedish; Swedish Medical Center; HCA HealthONE Swedish; HCA HealthONE Swedish; HCA HealthONE Swedish; HCA HealthONE Swedish; HCA HealthONE Swedish; HCA HealthONE Swedish; HCA HealthONE Swedish

## Abstract

**Introduction:**

The increasing demand for specialized burn care coupled with a shortage of qualified burn surgeons necessitates provider workforce optimization. Advanced Practice Providers (PAs and NPs) offer a solution to bridge this gap. This study investigates the impact of APP utilization in a non-academic burn center on surgical productivity and overall growth.

**Methods:**

A retrospective review of data from 2020 to 2023 was conducted at a non-academic burn and reconstruction center. The dataset encompassed surgical case volumes, patient admissions/clinic encounters, and provider employment data. Burn center growth was defined as the year-over-year percentage change in total patient encounters. Statistical analyses, including correlation, regression, and provider ratios, was employed to investigate the relationship between APP utilization, surgeon productivity, and burn center growth.

**Results:**

Increased APP utilization corresponded with a substantial rise in surgical productivity (68.8% overall growth) and patient encounter volume (28.4% overall growth). A strong positive correlation was observed between the number of APPs per surgeon and surgical productivity (r = 0.997, p < 0.001). A moderate positive correlation was also found between the number of APPs per surgeon and patient encounter volume growth (r = 0.632, p = 0.203). Regression analysis confirmed the significant predictive power of the APP-to-surgeon ratio on surgical productivity (F(2, 1) = 59.27, p = 0.036, R² = 0.969).

**Conclusions:**

The integration of APPs into a non-academic burn center’s care model demonstrates a promising approach to optimize workforce efficiency, enhance surgical productivity, and facilitate growth. By leveraging APPs, burn centers can improve their capacity to deliver efficient care to an expanding patient population.

**Applicability of Research to Practice:**

Non-academic burn centers lacking resident physician support can significantly benefit from incorporating APPs. Increased APP utilization has the potential to drive productivity gains and foster the growth of burn and reconstruction services.

**Funding for the Study:**

N/A

